# A Case Report of Caudal Migration of a Lumbar Epidural Catheter Confirmed by Doppler Ultrasound: Ultrasound to the Rescue!

**DOI:** 10.7759/cureus.55879

**Published:** 2024-03-10

**Authors:** Krishna U Chaitanya, DN Dhananjay, Mahendra K Kumar, Tulika Vinaik

**Affiliations:** 1 Department of Anaesthesiology, Sakra World Hospital, Bengaluru, IND

**Keywords:** postoperative analgesia, colour flow doppler, ultrasound, catheter migration, epidural failure

## Abstract

Combined spinal-epidural anaesthesia is an excellent technique for providing intraoperative and postoperative analgesia in patients undergoing total knee arthroplasty. Epidural catheters threaded through a Tuohy needle with a cephalad needle bevel orientation follow a winding pattern within the epidural space. Caudal or downward migration of an epidural catheter may lead to unsatisfactory anaesthesia and epidural failure. Colour flow Doppler sonography is emerging as an effective technique to determine the epidural catheter tip position. We report an interesting case of caudal migration of a lumbar epidural catheter confirmed by colour flow Doppler ultrasound.

## Introduction

Combined spinal-epidural anaesthesia is an excellent technique for providing intraoperative anaesthesia and postoperative analgesia in patients undergoing total knee arthroplasty. Failure of epidural anaesthesia has been reported to be about 13%-32% of all epidural catheter placements and can be due to a multitude of reasons [1-4]. An epidural catheter threaded through a Tuohy needle with a cephalad needle bevel orientation follows a winding pattern within the epidural space [3]. Imaging techniques have revealed that epidural catheters follow varied and unpredictable paths irrespective of the correct epidural space identification [5,6]. Hence confirmation of position of the catheter tip may lead to a decreased incidence of epidural failure. Several case studies employed colour flow Doppler sonography with varying results to determine catheter tip position [7-9].

We report the caudal migration of a lumbar epidural catheter confirmed by colour flow Doppler yet providing effective intraoperative anaesthesia and postoperative analgesia.

## Case presentation

A 64-year-old female weighing 72 kg and a height of 160 cm with a previous history of hypertension on antihypertensives was diagnosed with osteoarthritis of the knee joint. Preanaesthetic evaluation and routine blood investigation revealed no abnormalities with well-controlled blood pressure. The patient was posted for bilateral total knee arthroplasty under combined lumbar spinal epidural anaesthesia. On the day of surgery, the patient was shifted to the operating theatre, and standard monitors (pulse oximetry, electrocardiogram, and non-invasive blood pressure) were connected. A 20 GA IV cannula (BD Venflon ™) was secured on the dorsum of the left hand.

With the patient in a sitting position, a preliminary ultrasound (Mindray® MX7, Mindray Medical International Limited, China) of the spine was performed using a 2- 5 Hz curvilinear probe in both transverse and sagittal planes to identify the midline of the spine, lamina, anterior complex, posterior complex and distance from the skin to the posterior complex at the L3-L4. The distance from the skin to the posterior complex was determined to be 4.5 cm.

Under strict aseptic precautions, L3-L4 lumbar epidural space was identified by the loss of resistance to air technique using a Perican®- needle: 18 G x 3 ½” at around 5 cm. Lumbar puncture was done by needle through the needle technique with a Pencan®-needle: 27G x 5 3/8”. Free flow of CSF was seen and Bupivacaine Hydrochloride (Anawin™Heavy) 0.5% 2cc (10mg) was injected intrathecally. With the Huber’s tip directed upward, the epidural catheter (Perifix®-catheter:20G) was threaded through the Perican® Tuohy needle up to the 20cm mark, and the Perican® needle was removed.

**Figure 1:**
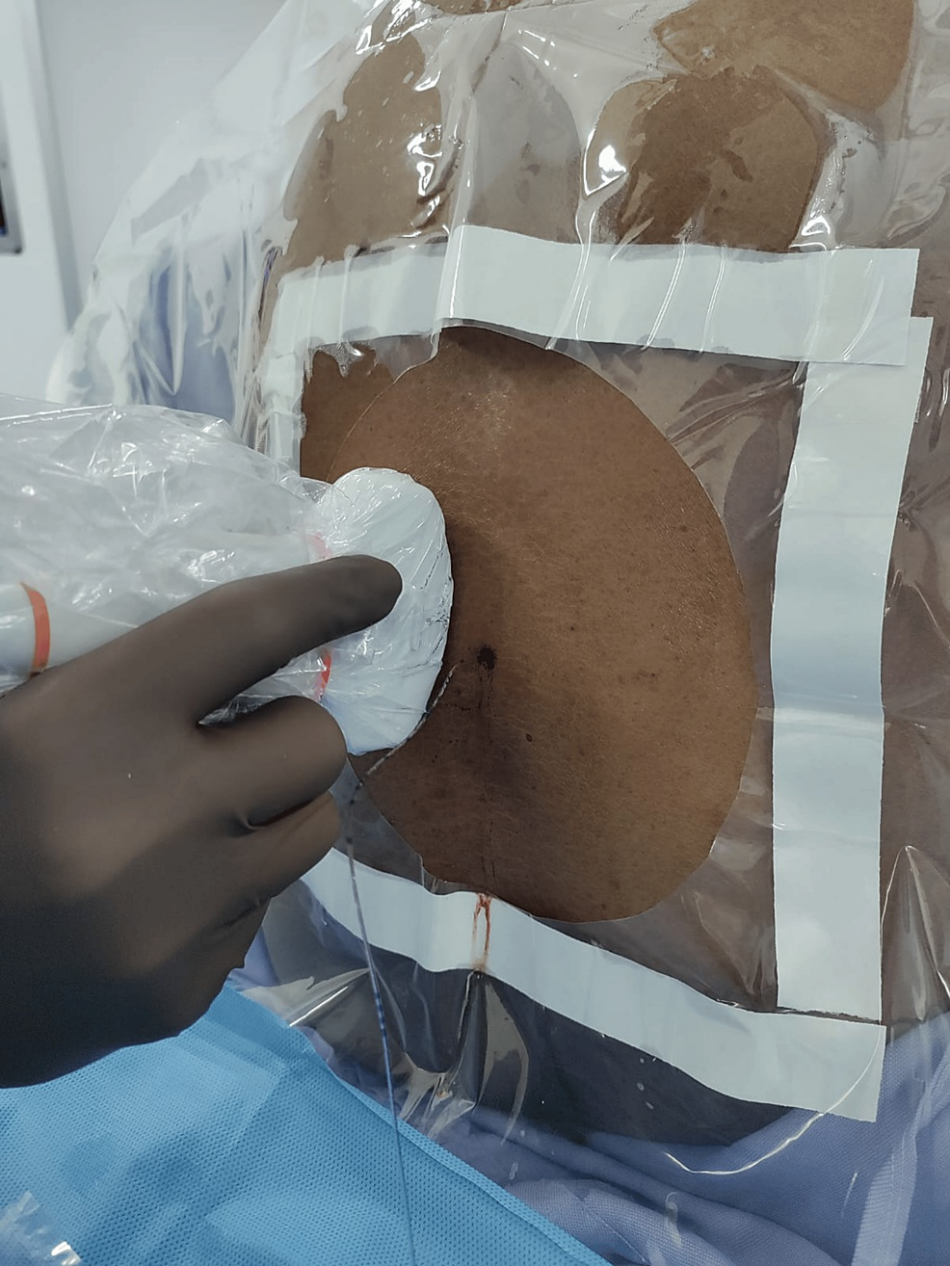
Ultrasound probe position to obtain para-sagittal oblique view of the spine

Colour flow Doppler was used to visualise flow through the tip of the epidural catheter in the para-sagittal oblique view by injecting short intermittent boluses of 0.1 mL of normal saline through the catheter using a BD® 1mL syringe (Figure 1).

**Figure 2:**
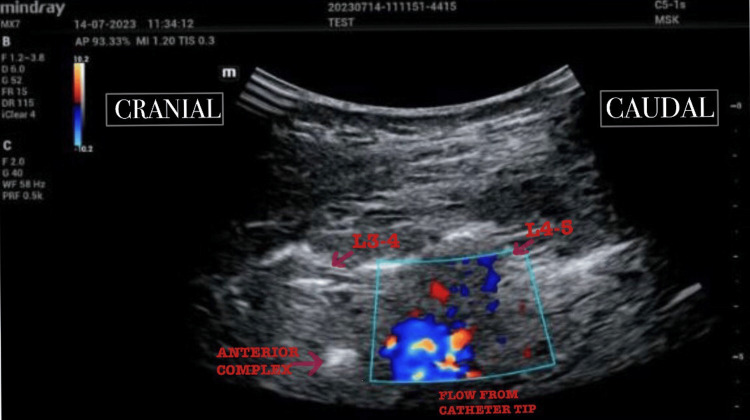
Flow from the catheter tip visualised in the L4 - L5 epidural space using colour flow Doppler

The assessment was initially performed in the L2 - L3 and L1 - L2 (2 levels above the site of insertion) lumbar intervertebral spaces. No flow was visualised in these epidural spaces. Subsequently, the assessment was performed in the L3 - L4 (level of epidural insertion), L4 - L5, and L5 -S1 intervertebral spaces (2 levels below the site of insertion), and flow was seen bilaterally on colour flow Doppler indicating caudal migration of the epidural catheter. The catheter was withdrawn 2 cm at a time and the assessment was repeated. Flow in the L5 - S1 epidural space was seen up to the 14 cm mark. Flow in the L4 - L5 and L3-L4 epidural spaces was seen up to the 10 cm mark (Figure 2).

The ultrasonographic examination was performed by a senior anaesthesiologist with experience in neuraxial ultrasound. The total time taken for the examination was around five minutes and the total time from injection of the spinal drug to the final supine position was 8- 10 minutes approximately. There was a chance of a saddle block with the longer duration of sitting position but considering the short height (160cm) of the patient the decision to inject 10 mg of 0.5% Bupivacaine Hydrochloride (Anawin™Heavy) intrathecally was taken to avoid any hemodynamic changes in the geriatric patient.

A detailed description of the technique of examination to obtain the relevant views is given by Elsharkawy et al. [8].

The catheter was fixed at the skin at 10 cm as per the institutional protocol of ensuring 5 cm of catheter length within the epidural space. The patient was made supine. The sensory level of the block was determined to be at the T10 dermatomal level and the motor block was complete (modified Bromage scale 1) [10] indicating adequate anaesthesia.

Intraoperative anaesthesia was maintained by a continuous epidural infusion of 0.5 % Bupivacaine Hydrochloride (Anawin™) at a rate of 20 mg (4 ml)/ hour starting one hour postepidural placement. The total surgical duration was four hours with adequate sensory (T10-T12 dermatomal level) and motor blockade (modified Bromage scale 1) [10]. As per institutional protocol, postoperative analgesia was maintained by a regimen of continuous epidural infusion of 0.1% Ropivacaine Hydrochloride (Ropin®) at the rate of 4 ml/hr, Paracetamol IV 1g q6h and Tramadol IV 50 mg q12h. Numerical rating scale scores on postoperative days 1, 2, and 3 were 3/10, 2/10, and 2/10 respectively.

## Discussion

Numerous methods of confirming epidural catheter placement have been reported including but not limited to electrical stimulation as described by Tsui et al. [11], interpretation of epidural pressure waveforms as described by Ghia et al [12], CT epidurography [13], contrast injection, and X-ray confirmation. These methods are technically difficult and require special equipment.

Ultrasonography has become a valuable tool for the modern anaesthesiologist in performing various procedures. One such application is ultrasound-guided epidural catheter insertion. Rapp et al. [7] demonstrated the epidural catheter position following epidural anaesthesia using ultrasonography in 18 of 25 pediatric patients studied and concluded that ultrasound was a valuable tool for epidural catheter placement in anaesthetised children. The study documented the level of epidural insertion but did not report the level of catheter tip position. In the retrospective study conducted by Elsharkawy et al. [8], the epidural catheter tip position was confirmed by Doppler ultrasound in 25 of the 37 patients investigated. The catheter tip was found to be one level below the site of epidural insertion in seven patients, but the effectiveness of postoperative analgesia in these patients was not reported. van den Bosch et al. [9] conducted a prospective study in 40 patients undergoing labour epidurals to assess the position of the catheter by visualizing flow with normal saline in the epidural space using colour flow Doppler. The study concluded that more than half of the epidural catheters remain at the interspace of insertion and additionally did not report flow caudal to the level of insertion in any of the patients. We report a case of a caudally migrated epidural catheter successfully confirmed by colour flow Doppler. Despite caudal migration, the epidural catheter effectively provided both intraoperative anaesthesia to facilitate the surgical procedure and satisfactory postoperative analgesia considering the fact the surgical procedure was on the lower limb. It may be valuable to correctly identify epidural catheter position, especially in surgical procedures involving the abdomen as caudal migration of epidural catheters may result in inadequate analgesia.

The colour flow Doppler technique of identifying epidural catheters has a few limitations. There is a learning curve required to obtain the relevant ultrasonographic views. In addition, the pattern of flow obtained in failed epidural catheters requires further multicentric studies.

## Conclusions

The technique of epidural catheter placement has been refined to a great degree by the use of ultrasonography. It has evolved from a blind, landmark-guided technique to a precise image-guided technique due to accurate real-time visualization of spine anatomy. Colour flow Doppler is a simple, non-invasive, widely available technique of catheter confirmation with an easy learning curve that may lead to a decreased incidence of failed epidurals. Our case highlights the importance of determining the path catheters take within the epidural space as caudal migration may result in inadequate and ineffective anaesthesia. It is of utmost importance to prevent this phenomenon before initiating a surgical procedure and not as an afterthought. 
